# Association of Vitamin D Deficiency and Newly Diagnosed Pulmonary Tuberculosis

**DOI:** 10.1155/2021/5285841

**Published:** 2021-01-15

**Authors:** Vijay Jaimni, Barkur Ananthakrishna Shasty, Sharath P. Madhyastha, Ganesh V. Shetty, Raviraja V. Acharya, Ragini Bekur, Akhila Doddamani

**Affiliations:** ^1^Department of Medicine, Kasturba Medical College, Manipal Academy of Higher Education, 576104, Manipal, Karnataka, India; ^2^Department of Community Medicine, Kasturba Medical College, Manipal Academy of Higher Education, 576104, Manipal, Karnataka, India

## Abstract

**Introduction:**

Vitamin D has a significant role in host immune defense against Mycobacterium tuberculosis. It has been suggested that pulmonary tuberculosis may be associated with lower levels of vitamin D. Present study was therefore undertaken to identify the association between vitamin D deficiency and pulmonary tuberculosis.

**Methods:**

A case-control study was conducted in a tertiary care hospital from 2014 to 2016, including 50 adult newly diagnosed sputum positive pulmonary tuberculosis patients as cases and 50 age and sex-matched healthy participants as control groups. All participants in the study group had undergone detailed clinical examination and routine laboratory investigations, including vitamin D, calcium, and sputum for AFB. The clinical characteristics, X-ray findings, sputum AFB, and vitamin D levels were analyzed and compared with data obtained from healthy controls.

**Results:**

In both groups, the majority were men (88%). BMI was significantly (<0.0001∗) lower in the tuberculosis group (19.40 (17.20, 22.0) vs. 24.00 (22.50, 25.47)). Serum vitamin D levels were significantly lower (*P* = 0.012) in the tuberculosis group (19 (7.75, 27.25) ng/dl) as compared to the control group (25 (19.75, 32.00) ng/dl). Out of 50 TB patients, 27 (54%) had vitamin D deficiency, while among healthy controls, only 13 (26%) had vitamin D deficiency. Among vitamin D deficient PTB patients, 44% had 3+/hpf AFB in sputum smear examination.

**Conclusion:**

The prevalence of vitamin D deficiency in pulmonary tuberculosis cases is very high. Hypovitaminosis D was associated with more severe clinical symptoms, higher sputum smear positivity, and extensive lesions in chest radiograph among pulmonary tuberculosis patients.

## 1. Introduction

Vitamin D or calciferol is a fat-soluble vitamin that has a significant clinical role in calcium homeostasis and bone metabolism. Vitamin D from the skin and diet is metabolized in the liver to 25-hydroxyvitamin D (25(OH)D), which is used to determine the patient's vitamin D status. This 25(OH)D is converted to its active form, 1,25-dihydroxy vitamin D (1,25(OH)2 D) in the kidneys by the enzyme 25-hydroxyvitamin D-1*α*hydroxylase [1]. Vitamin D has a vital role in host immune defense against *Mycobacterium tuberculosis* (MTB). It has been observed that vitamin D induces antimicrobial peptide cathelicidin which inhibits mycobacterium multiplication in the macrophages [2, 3]. High serum vitamin D concentrations and hypercalcemia have been described in patients with tuberculosis. Activated macrophages in granulomas are the most likely source of the high calcitriol levels [4, 5]. But many studies worldwide have suggested that pulmonary tuberculosis (PTB) is associated with lower levels of vitamin D [6–8]. Also, few studies showed no significant difference between the mean serum calcium and vitamin D level between the PTB patients and the controls [9, 10]. Present study was therefore undertaken to identify the relation between vitamin D levels and PTB.

## 2. Materials and Methods

The present study was a case-control study carried out in a tertiary care hospital, Manipal, India, from October 2014 to April 2016, which included 100 participants. Participants were split into 2 groups. The first group (study group) comprised 50 newly diagnosed sputum smear-positive PTB patients admitted to general medicine and pulmonary medicine departments. Group 2 (control group) formed 50 age and gender-matched healthy controls enrolled from the general population that included relatives of admitted patients and health care workers with the similar ethnic and socio-economic background as that of the study group. PTB was ruled out in the control group by a thorough history and clinical examination. Approval from the Institutional Ethics Committee (IEC 631/2014) was sought and granted. Written informed consent was taken from all the participants.

### 2.1. Inclusion Criteria

All patients aged more than 18 years with newly diagnosed sputum smear-positive pulmonary tuberculosis were included in the study group.

### 2.2. Exclusion Criteria

Participants with self-reported diseases like human immune deficiency (HIV) virus infection, diabetes mellitus, cancer; patients who were on corticosteroids and chemotherapeutic drugs; and patients with extrapulmonary tuberculosis, pregnancy or lactating mothers, sarcoidosis, and parathyroid disorders were excluded. Patients who were on vitamin D, calcium supplements, anticonvulsants, diuretics, or any other drug which interacts with vitamin D and patients with chronic liver or renal disease, malabsorption conditions, gastric, or bowel resection were also excluded from the study.

All participants underwent a detailed medical history and clinical examinations. Routine lab investigations like complete blood count, liver and kidney function tests, blood glucose levels, HIV ELISA, HBsAg and anti-HCV serology, serum calcium, and albumin levels were done in all participants. Chest radiograph and at least two baseline early morning sputum samples were obtained from each patient for acid-fast staining, as per the Revised National Tuberculosis Control Program (RNTCP) guidelines [11].

Serum 25(OH)D level was measured before initiating antitubercular therapy (ATT). Although 1,25(OH)2D is the biologically active form of vitamin D, the ideal marker for vitamin D deficiency is 25(OH)D. Because 25(OH)D is the major circulating form of vitamin D and circulating concentration of 1,25(OH)2D is 1000th the concentration of 25(OH)D. The half-life for 1,25(OH)2D is very less compared to 25(OH)D. Finally, as a person becomes vitamin D deficient and develops secondary hyperparathyroidism, the renal 1-OHase generates more 1,25(OH)2D. Thus, when a patient is vitamin D insufficient, there is often a normal or even elevated blood level of 1,25(OH)2D. Hence, the measurement of 25(OH)D is the only method to determine vitamin D deficiency [12]. As per the Endocrine Society clinical practice guidelines, 25(OH)D levels were divided into three categories [1]. 25(OH)D level ≤ 20 ng/ml was considered as deficient, 25(OH)D level 21–29 ng/ml as insufficient, and 25(OH)D level more than 30 ng/ml was considered as optimal. The serum 25(OH)D concentration was measured by electrochemiluminescence immunoassay (ECLIA). The analyzer used was Beckman coulter Access-2, manufactured in California, USA, and the quality control (QC) measures were instituted by BIORAD (both internal and external QC). Analyzer reference range of serum 25(OH)D level is 2.0–167 ng/ml OR 5.0–417 nmol/l. According to WHO definition, “hemoglobin level less than 12 gm/dl in women and less than 13 gm/dl in men is considered as anemic.” [13] Hypoalbuminemia was defined as a total serum albumin level less than 3.5 g/dl while hypocalcaemia as corrected serum calcium concentration less than 9 mg/dl [14].

The corrected serum calcium concentration for the albumin levels was calculated using following formula, “Corrected total calcium (mg/dL) = 0.8 x [4 − serum albumin (g/dL)] + Total calcium (mg/dL).” [15]

Based on chest X-ray findings, tuberculosis was categorized into two categories [16–18].

1. Primary pulmonary tuberculosis (PPT) includes parenchymal disease, typically manifests as dense, homogeneous parenchymal consolidation predominantly in the lower and middle lobe (subpleural sites), lymphadenopathy, and pleural effusion
2. Progressive pulmonary tuberculosis (PrPT) includes parenchymal disease as patchy consolidation (typically in the upper lobe), cavitatory lesion, miliary tuberculosis, and multiple mediastinal lymphadenopathies

Based on sputum smear findings, tuberculosis was grouped into three categories [2, 19].

1. +++ per hpf. (3+/high power field)
2. ++ per hpf
3. + or scanty per hpf

Patients who were found to have vitamin D deficiency were given a therapeutic dose of vitamin D as per the recommendations [1].

### 2.3. Statistical Analysis

Data analysis was carried out using the SPSS software 20.0. Results are presented as median and interquartile range (IQR). Continuous variables were analyzed using Mann–Whitney *U* test. Associations between the categorical variables were analyzed by using Fisher's exact test or chi-squared test. The statistical significance was set at *P* ≤ 0.05.

## 3. Results

A total of 100 participants were included in the study, out of whom 50 were newly diagnosed sputum smear-positive PTB and 50 were age and sex-matched healthy controls. In both groups, the majority were men (88%). There was no significant variation in age and sex between the two groups. BMI (body mass index) was significantly (*P* < 0.0001∗) lower in the tuberculous group as compared to healthy controls. There was a significantly higher proportion of smokers (*n* = 19 (38%)) among the PTB cases compared to controls (*n* = 10 (20%)). Vitamin D levels were significantly lower in the tuberculosis group (19 (7.75, 27.25)) than in the control group (25 (19.75, 32.00)) (Table 1).

Out of 50 TB patients, 27 (54%) had vitamin D deficiency, and 18% had sufficient levels. While among healthy controls, only 13 (26%) had deficient vitamin D levels. The difference is statistically significant (*p*-0.004) (Table 2).

Tuberculosis was categorized into PPt and PrPt based on X-ray findings. In the present study, out of 50 patients with pulmonary tuberculosis, 27 were found to have deficit vitamin D levels; in the deficient category, 7 (26%) had PPt while 20 (74%) had PrPt. Out of 14 patients with insufficient vitamin D levels, 3 (21%) had PPt, while 11 (78%) had PrPt (Table 3).

In the present study, out of 50 patients with pulmonary tuberculosis, 27 had deficient vitamin D levels, out of whom 12 (44%) had 3+ AFB/hpf in sputum smear examination, 11 (41%) had 2+ AFB/hpf, and 4 (15%) had 1+ AFB/hpf (Table 4).

## 4. Discussion

### 4.1. Vitamin D Levels in Tubercular Patients and Healthy Controls

Vitamin D, a fat-soluble vitamin, has a vital role in host immunity against *Mycobacterium tuberculosis*. Calcitriol is the biologically active form of vitamin D, which leads to the expression of a peptide called cathelicidin. This cathelicidin is a microbicidal peptide that acts against *Mycobacterium tuberculosis* [3]. It has been illustrated that vitamin D supplementation leads to augmented expression of cathelicidin peptide in the macrophages and thereby boosting innate immunity in patients with tuberculosis [20].

The present study conducted on newly diagnosed sputum positive pulmonary tuberculosis patients and healthy controls showed that the prevalence of vitamin D deficiency was significantly higher in patients with tuberculosis than the healthy group. In the tuberculosis group, 27 (54%) patients were found to have a deficit in vitamin D compared to 13 (26%) in the healthy control groups.

Many studies were done from various continents suggesting vitamin D levels are low in patients with pulmonary tuberculosis [21–24]. A case-control study in Vietnam, which involved 166 TB patients and 219 controls, showed that the prevalence of vitamin D insufficiency was 35.4% in men with tuberculosis compared to 19.5% in controls (*P* = 0.01) [21]. Other studies done on African populations in Guinea-Bissau [22], Asian immigrant population of Gujarati origin, residing in UK [23], and African people living in Melbourne [24] also reported that vitamin D levels were significantly reduced in tuberculosis patients. Even in India, a case-control study done by Sashidharan et al. in Kerala observed very low levels of vitamin D in tuberculosis patients compared to healthy controls [25].

### 4.2. Clinical Symptoms in Hypovitaminosis D and Tuberculosis

Hypovitaminosis D can present in tuberculosis patients with nonspecific symptoms such as bone and muscle pain, arthralgia, and tiredness. These clinical features are often present in multiple disease entities such as connective tissue diseases, chronic fatigue syndrome, fibromyalgia, or even depression. Hence, the diagnosis may be delayed in many cases [26].

In the present study, 40% of the tuberculosis patients who were found to be vitamin D deficient presented with myalgia and bone pain. 70% of the patients in this group complained of generalized weakness. In the healthy group, 30% of the controls who were deficient in vitamin D levels presented with bone pain and myalgia, and 50% complained of generalized weakness.

Chronic severe vitamin D deficiency in adults may lead to proximal muscle weakness, bone pain, and osteomalacia. Less severe vitamin D inadequacy leads to elevation of PTH (secondary hyperparathyroidism), leading to increased bone demineralization, bone loss, and finally osteoporosis and fracture [27].

### 4.3. Low Vitamin D Levels and Degree of Sputum Smear Positivity

A cross-sectional study done by Yuvaraj et al. [2] in India, which included 65 sputum AFB positive PTB patients and 65 healthy controls, showed a significant difference in the deficiency of vitamin D with a rise in sputum smear positivity associated with a substantial negative correlation between vitamin D levels and degree of sputum smear positivity. The study concluded that the lower the vitamin D, the higher the MTB bacterial load [2]. In the present study also, we observed a significant negative correlation between vitamin D levels and degree of sputum smear positivity and levels of sputum positivity.

### 4.4. Low Vitamin D Levels and Chest X-Ray Findings in Tuberculous Patients

In the present study, based on chest X-ray findings, out of the 27 patients with vitamin D deficiency, 74% (20) had progressive pulmonary tuberculosis, and only 25% (7) had primary pulmonary tuberculosis. The patients with deficient vitamin D levels were found to have bilateral and extensive lesions in the radiological evaluation of X-ray chest.

A randomized, double-blinded, multicentric study done by Salahuddin et al. [28] revealed the negative correlation between vitamin D levels and the severity of chest X-ray involvement. They also reported that the correction of vitamin D deficiency accelerated the clinical and radiographic improvement in all patients with tuberculosis [28].

A randomized controlled trial done by Khandelwal et al. [29] included 266 children with intrathoracic tuberculosis found that more than half of subjects had progressive pulmonary TB (56%). The remaining participants belonged to primary pulmonary complex (30.5%) and pleural effusion (13.5%). They have also found that only 9% had sufficient levels of vitamin D [29].

### 4.5. The Relation between Vitamin D Levels and Severity of Pulmonary Tuberculosis

In our study, the patients with low vitamin D levels had a higher degree of sputum smear positivity and more extensive lesions in chest radiograph suggestive of more severe tuberculosis.

A cross-sectional study by Rathored et al. included 354 MDR-TB stated that MDR-TB patients have significantly lower levels of vitamin D and serum calcium. They also reported that there was delayed smear conversion in patients with vitamin D deficiency [30]. Tuberculosis itself can lead to paradoxical depletion of vitamin D metabolism during therapy, thus linking the severity of the disease [31].

### 4.6. Limitations of the Study

1. In the present study, an adequate sample size was not achieved as the survey was time-bound. Hence, it is difficult to generalize the study findings. Further prospective studies with larger sample size are required to understand the relation between vitamin D and tuberculosis
2. There may be differences in UV exposure among study participants and its possible role in serum vitamin D deficiency
3. Patients were given a therapeutic dose of vitamin D, and they were not followed up. Further studies are required to study vitamin D's role in the prognosis and outcome of pulmonary tuberculosis

## 5. Conclusion

In the present study, vitamin D deficiency is highly prevalent among patients with pulmonary tuberculosis compared to healthy individuals. Hypovitaminosis D is associated with more severe clinical symptoms in patients with tuberculosis. Vitamin D deficiency is associated with a higher degree of sputum smear positivity and more extensive lesions in chest radiograph among pulmonary tuberculosis patients.

## Figures and Tables

**Table 1:** Determinants of pulmonary tuberculosis.

Variables	Group 1 (*n* = 50) (tuberculosis)	Group 2 (*n* = 50) (control)	*P* value
BMI (kg/m^2^) (median, IQR)	19.40 (17.20, 22.0)	24.00 (22.50, 25.47)	<0.0001^∗^
Serum 25(OH)D (ng/dl) (median, IQR)	19 (7.75, 27.25)	25 (19.75, 32.00)	0.012^∗^
Serum albumin (median, IQR)	3.18 (2.70, 3.70)	4.30 (3.94, 4.50)	<0.0001^∗^
Corrected serum calcium (median, IQR)	9 (8.67, 9.20)	9.20 (8.98, 9.50)	0.18

Mann–Whitney *U* test, ^∗^*P* ≤ 0.05 (statistical significance). BMI: body mass index; 25(OH)D: 25-hydroxyvitamin D.

**Table 2:** Vitamin D levels among pulmonary tuberculosis patients (*N* = 50) and controls (*N* = 50).

Vitamin D levels		Tuberculosis patients *n* (%)	Healthy controls *n* (%)	*p* value
Deficient (< 20 ng/ml)	Present	27 (54%)	13 (26%)	0.004^∗^
	Absent	23 (46%)	37 (74%)	
Insufficient (20-30 ng/ml)	Present	14 (28%)	20 (40%)	0.205
	Absent	36 (72%)	30 (60%)	
Sufficient (> 30 ng/ml)	Present	9 (18%)	17 (34%)	0.068
	Absent	41 (82%)	33 (66%)	

Chi-squared test, ^∗^*P* ≤ 0.05 (statistical significance).

**Table 3:** Vitamin D level in primary pulmonary tuberculosis and progressive pulmonary tuberculosis.

Vitamin D levels	Primary pulmonary tuberculosis *N* (%)	Progressive pulmonary tuberculosis *N* (%)	*p* value
Deficient (< 20 ng/ml) (*n* = 27)	7 (25.93%)	20 (74.07%)	0.0009^∗^
Insufficient (20-30 ng/ml) (*n* = 14)	3 (21.43%)	11 (78.57%)	0.007^∗^
Sufficient (> 30 ng/ml) (*n* = 9)	1 (10%)	8 (90%)	0.003^∗^

Fisher's exact test, ^∗^*P* ≤ 0.05 (statistical significance).

**Table 4:** Comparison of vitamin D levels with sputum findings in pulmonary tuberculosis.

Vitamin D levels	Sputum smear AFB density			*P* value
	3 + *N* (%)	2 + *N* (%)	1 + or scanty *N* (%)	
Deficient (< 20 ng/ml) (*n* = 27)	12 (44%)	11 (41%)	4 (15%)	0.046^∗^
Insufficient (20-30 ng/ml) (*n* = 14)	5 (36%)	6 (42%)	3 (22%)	0.601
Sufficient (> 30 ng/ml) (*n* = 9)	3 (33%)	2 (22%)	4 (44%)	0.873

Fisher's exact test, ^∗^*P* ≤ 0.05 (statistical significance).
